# Supplementary figures and images for: Parcellating connectivity in spatial maps
Source: PeerJ. 2015 Feb 19;3:e784. doi: 10.7717/peerj.784 (PMC4338796; doi:10.7717/peerj.784)

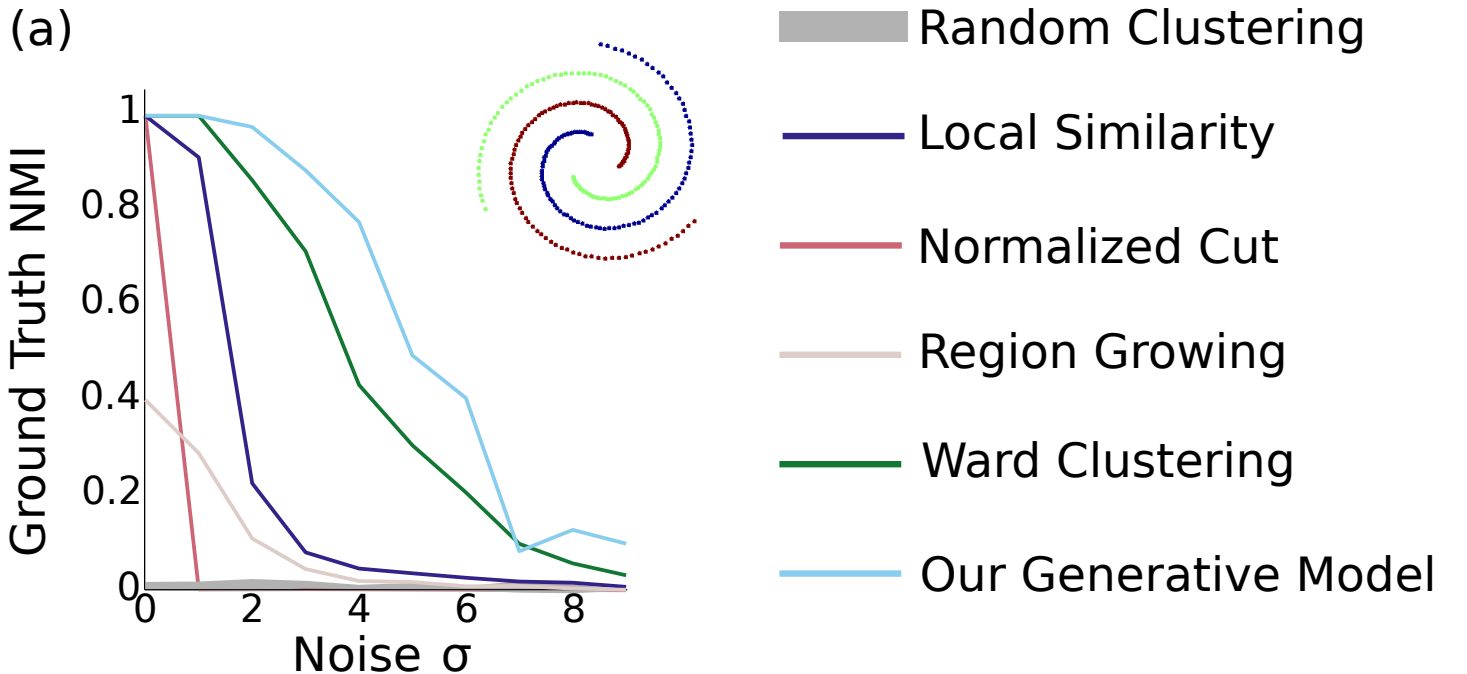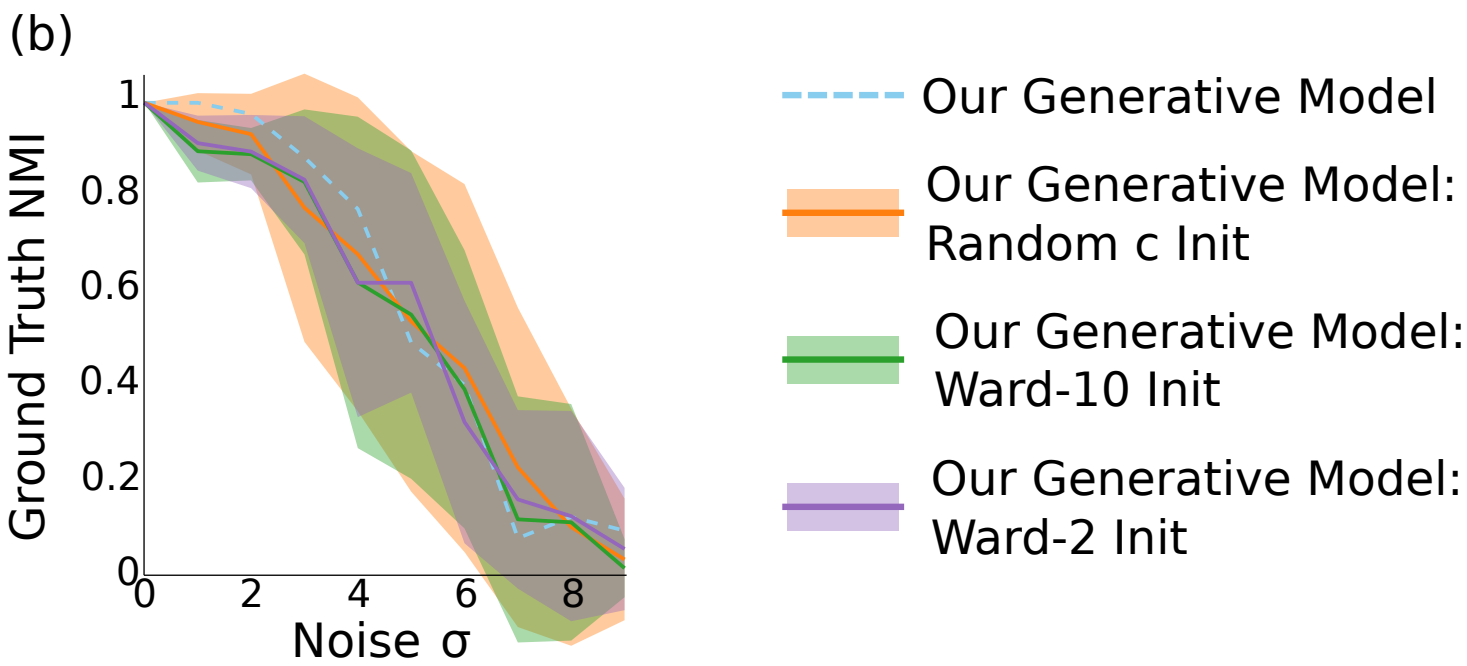

Supplement: Figure S1 — (A) We evaluated our method on a challenging three-spiral dataset, as described in the main text. As in our other three synthetic data experiments, we outperform all comparison methods, and show a substantial improvement over greedy Ward clustering at all but the highest noise levels. (B) The precise initialization scheme used for our method does not have a substantial impact on the results. Using a Ward initialization with a fixed number of clusters (2 or 10) rather than using our model to pick the most likely initialization, or using an entirely random initialization, all yield similar performance (shaded regions shows the standard deviation across randomly generated connectivities at each noise level). The random initialization did require more passes over the data to reach this level of performance (1,000 vs. 100 for other initializations). [file peerj-03-784-s001.pdf]
